# A Literature Review of the Potential Diagnostic Biomarkers of Head and Neck Neoplasms

**DOI:** 10.3389/fonc.2020.01020

**Published:** 2020-06-26

**Authors:** Heleen Konings, Sofie Stappers, Margot Geens, Benedicte Y. De Winter, Kevin Lamote, Jan P. van Meerbeeck, Pol Specenier, Olivier M. Vanderveken, Kristien J. Ledeganck

**Affiliations:** ^1^Faculty of Medicine and Health Sciences, University of Antwerp, Antwerp, Belgium; ^2^Laboratorium of Experimental Medicine and Pediatrics and Member of the Infla-Med Centre of Excellence, University of Antwerp, Antwerp, Belgium; ^3^Department of Pneumology, Antwerp University Hospital, Edegem, Belgium; ^4^Department of Internal Medicine and Pediatrics, Ghent University, Ghent, Belgium; ^5^Department of Oncology, Antwerp University Hospital, Edegem, Belgium; ^6^Center for Oncological Research (CORE), University of Antwerp, Antwerp, Belgium; ^7^Department of Otorhinolaryngology-Head and Neck Surgery, Antwerp University Hospital, Edegem, Belgium; ^8^Department of Translational Neurosciences, Antwerp University, Antwerp, Belgium

**Keywords:** head and neck neoplasms, biomarker, genomics, proteomics, metabolomics, volatomics, microbiomics, radiomics

## Abstract

Head and neck neoplasms have a poor prognosis because of their late diagnosis. Finding a biomarker to detect these tumors in an early phase could improve the prognosis and survival rate. This literature review provides an overview of biomarkers, covering the different -omics fields to diagnose head and neck neoplasms in the early phase. To date, not a single biomarker, nor a panel of biomarkers for the detection of head and neck tumors has been detected with clinical applicability. Limitations for the clinical implementation of the investigated biomarkers are mainly the heterogeneity of the study groups (e.g., small population in which the biomarker was tested, and/or only including high-risk populations) and a low sensitivity and/or specificity of the biomarkers under study. Further research on biomarkers to diagnose head and neck neoplasms in an early stage, is therefore needed.

## Introduction

Head and neck cancers account for 5% of all malignant tumors and are responsible for about 600,000 new cases and 300,000 deaths in the world annually. About 50% of the patients fail to achieve cure and cancer relapse occurs despite intensive combined treatment (1, 2). To date, there is no adequate biomarker available for the diagnosis of head and neck cancer. However, it is expected that an earlier detection could improve the patient's outcome stage (3–7). In this review, we provide a general overview of biomarkers that were investigated to diagnose head and neck neoplasms in an early phase. Besides, we go into detail on the restrictions of these candidate biomarkers in the clinical practice.

Head and neck neoplasms are defined as benign, premalignant and malignant tumors above the clavicles, with exception of tumors of the brain, and spinal cord and esophagus (2). This includes tumors of the paranasal sinus, the nasal cavity, the salivary glands, the thyroid, and the upper aerodigestive tract (oral cavity, pharynx, and larynx) (8, 9). Carcinomas of the head and neck preferably metastasize lymphogenic to the regional lymph nodes and it is only in an advanced stage that they metastasize hematogenic to the lungs, liver, and bones (1). The histopathology of the cancers differs from site to site, but the most common ones are squamous cell carcinomas, accounting for more than 85% of the head and neck neoplasms (8, 9).

There are several known risk factors for the development of head and neck carcinomas. Prolonged exposure to the sun has been shown to be partly responsible for the genesis of skin and lip cancer (2). Epstein-Barr virus infection, living in a smoky environment and eating raw salted fish are important risk factors in the development of a nasopharyngeal carcinoma (1, 8). Infection with human papilloma virus plays a significant role in the genesis of oropharyngeal cancer (8). Carcinomas of the paranasal sinuses are more frequently seen in woodworkers; particularly tropical hardwood forms an important trigger (1). Chewing betel nut, especially in Asia, plays a major role in the etiology of cancer of the oral cavity (1). Excessive use of tobacco and alcohol induces mucosal changes of the aerodigestive tract. These alterations play an important role in the genesis of malignant tumors (1, 8). Besides tobacco and alcohol, other factors influence mucosal changes like nutritional deficiencies (in particular vitamin A and vitamin C in the context of insufficient intake of fresh fruits and vegetables) and genetic predisposition (1, 8). In the development of tumors of the skin, mucosa, thyroid gland, parathyroid glands and the salivary glands, former exposure to ionizing radiation might also be of influence (2).

In comparison with other malignant tumors, head, and neck neoplasms are not common in the Western world. However, a rapid increase of the incidence of oropharyngeal cancers related to HPV in developed countries, has been shown (8). Although this incidence (and therefore the mortality rate) is lower compared to other cancers, patients with head and neck cancer have a poor prognosis, mainly due to the fact that these types of cancers are usually diagnosed at an advanced stage (3–7). One-third of the patients gets medical care in an early stage, while two-third are only diagnosed when they already entered an advanced stage (1). According to the WHO, the most common sites of head and neck neoplasms are the oral cavity, the larynx and the pharynx (8). The highest incidence of head and neck cancer is seen in South East Asia (8). Head and neck neoplasms are more prevalent in men than women and they most likely appear in the age range of 60–80 years. The average age of diagnosis is 62 years for men and 63 years for women (1).

## Methods

**Figure 1:**
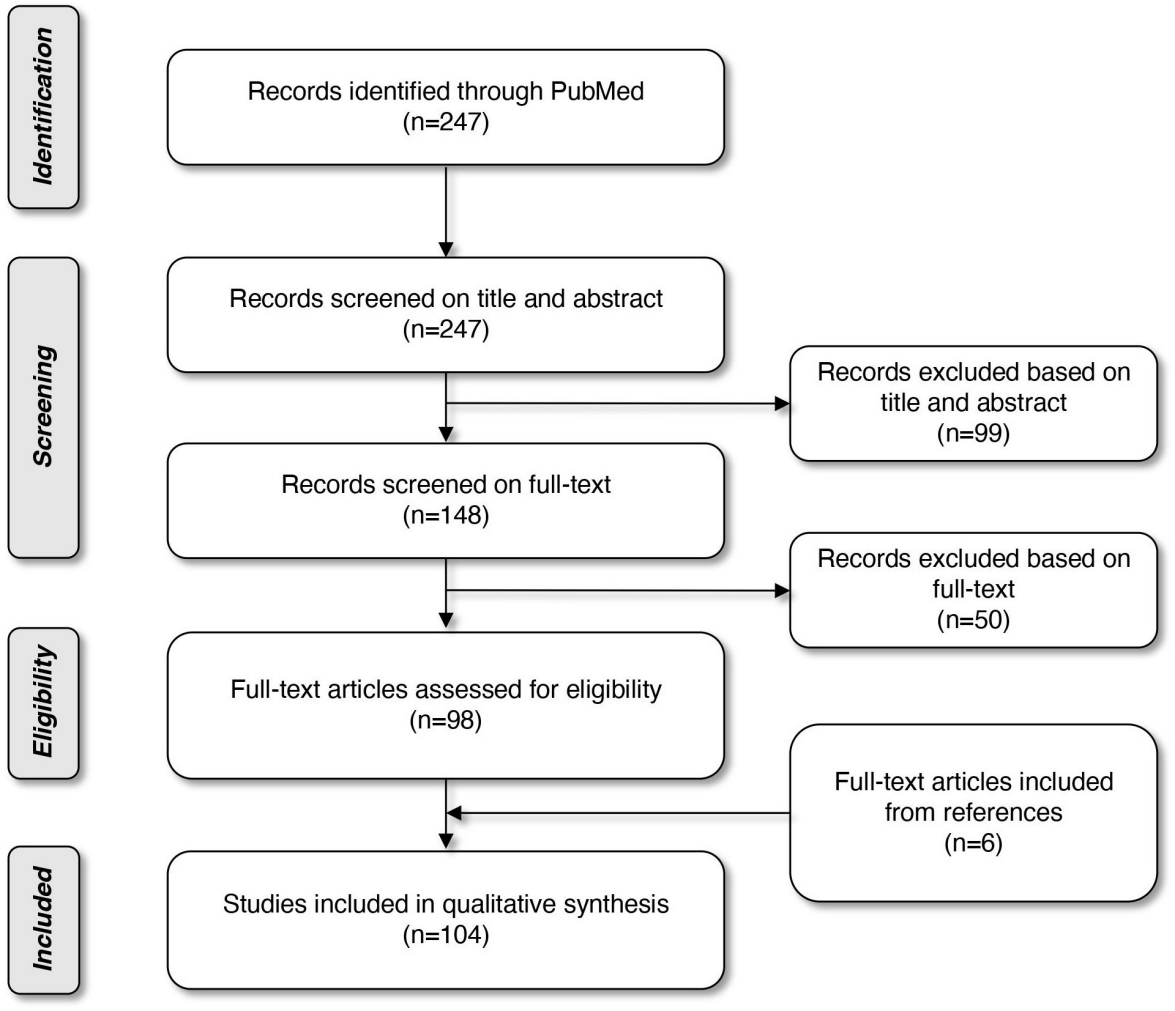
Flow diagram of article selection.

Literature was searched through MEDLINE (PubMed Database). The search started in October 2017 and was limited to papers published in the last decade. The last database search was performed on March 31st, 2019. A combination of the following Mesh terms was used: “biomarkers, tumor”; “head and neck neoplasms”; “early diagnosis”; “volatile organic compounds”; “microbiota”; “papillomaviridae”; “radiomics,” leading to 247 articles. Based on the title and abstract, we selected 148 articles and after reading the full texts and assessing the quality of the texts, we eventually ended up with 102 articles on this topic. The quality of the studies was assessed by three researchers (SS, HK, and MG). To broaden our search and complete our electronic query, reference lists of articles already withheld, were also verified. Six more articles were included through this snowball method. In total, 108 articles were included in this review. The selection procedure is displayed in a flow diagram (Figure 1).

## Results

Since head and neck cancers have a poor prognosis and are most frequently diagnosed at an advanced stage, finding a biomarker for early diagnosis of these tumors, is of tremendous importance to reduce morbidity and mortality (3, 10). Therefore, several biomarkers have been investigated so far. We provide a general overview of known biomarkers for diagnosis of head and neck neoplasms, including a discussion of the most promising markers. Strictly speaking, cancers of the upper part of the esophagus are also part of the head and neck tumors. In this literature review, however, they were not included. In Tables 1–8, all the investigated diagnostic markers with brief additional information are presented. More details about the biomarkers with their restrictions and advantages are provided in the Supplementary Tables 1–5. In the following paragraphs, we will discuss and present the biomarkers based on the applied -omics approach (genomics, proteomics, metabolomics, glycomics, volatomics, microbiomics, and radiomics).

**Table 1:** Summary of genomics in head and neck neoplasms.

**Studied marker**	**Type of tumor**	**No. of patients**	**Sample type**	**Expression**	**Test accuracy indices**			**References**
					**Sens**.	**Spec**.	**Accuracy**	
**(PROMOTER) HYPERMETHYLATION**								
Promoter hypermethylation panel (HOXA9 and NID2)	OSCC	179	Tissue biopsy, salivary rinses	↓	Tissue			(3)
					94%	75%	–	
					Saliva			
					75%	53%	–	
Methylation of cg01009664 of the thyrotropin-releasing hormone (TRH) gene	OSCC and OPSCC	89	Oral rinse and swab	↓	Swab			(11)
					91.30%	84.85%	–	
					Rinse			
					86.15%	89.66%	–	
Promoter hypermethylation panel (PTEN and p16)	OSCC	50	Tissue biopsy	↓	–			(12)
EBV DNA load + hypermethylation panel (RASSF1A, WIF1, DAPK1, and RARβ2)	NPC	252	Tissue biopsy, NP brushing, cell-free plasma	↓/↑	EBV DNA positive and hypermethylation panel in cell-free plasma			(13)
					–	88%	–	
					Hypermethylation panel in NPC biopsies and NP brushings			
					95.8%	67.4%	–	
**EBV RELATED MARKERS**								
IgA VCA + EBV DNA load	NPC	139	Blood sample	↑	99%	96–98%	–	(14)
**miRNA**								
Combination of miR-196a and miR-196b	Oral cancer	90	Blood sample	↑	88%	93%	–	(15)
Three-plasma miRNA panel (miR-222-3p, miR-150-5p, and miR-423-5p)	OL, OSCC	250	Blood sample	↑	–			(16)
miR-331-3p, miR-603, miR-1303, miR-660-5p, and miR-212-3p individually	LSCC	20	Blood sample	↑	–			(7)
Combination of hsa-miR-657 and hsa-miR-1287	Larynx carcinoma	39	Tissue biopsy	↓/↑	86.21%	100%	–	(15)
miR-155	LSCC	280	Tissue biopsy, blood sample	↑	58.4%	69.5%	–	(17)
**INTERFERONS**								
Interferon inducible transmembrane protein 1 (IFITM1)	OSCC	38	Tissue biopsy	↑	–			(18)
ISG15	OSCC	30	Tissue biopsy	↑	–			(19)
**ANTIGENS**								
Melanoma associated antigens-A (MAGE-A)	OSCC	70	Brush biopsy	↑	–			(20)
**UPREGULATED GENES**								
ADAM15, CDC7, IL12RB2, and TNFRSF8	OSCC	33	Tissue biopsy	↑	ADAM15			(21)
					52.9%	82.4%	–	
					CDC7			
					52.9%	82.4	–	
					IL12RB2			
					35.5%	94.1%	–	
					TNFRSF8			
					35.5%	88.2%	–	
**HPV RELATED MARKERS**								
HPV-16 E6 antibodies	OPC (HPV driven (*n* = 5) and non-HPV driven [*n* = 4)]	9	Blood sample	Seropositive	HPV-driven OPC			(22)
					100% (95% CI = 48–100%)	–	–	
					Non-HPV driven OPC			
					–	100% (95% CI = 40–100%)	–	
HPV-16 E6 antibodies	OPSCC (HPV driven)	63	Blood sample	Seropositive	96% (95%CI = 88–98%)	98 (95% CI = 90–100%)	97% (95% CI = 92–99%)	(23)
HPV-16 E6 antibodies	HNSCC (excluding OPC)	3	Blood sample	Seropositive	50% (95% CI = 19–81%)	100% (95% CI = 96–100%)	97% (95% CI = 91–99%)	(23)
HPV-16 E7	HNSCC (HPV driven)	30	Blood sample	Seropositive	–	–	–	(24)
			Saliva sample		–	–	–	
**OTHERS**								
5-hydroxylmethylcytosine (5-hmC)	OSCC	23	Tissue biopsy	↓	–			(25)
Total cfDNA	HNSCC, especially OPSCC	27	Blood sample	↑	–			(26)

*EBV, Epstein-Barr virus; HNSCC, head and neck squamous cell carcinoma; HPV, human papillomavirus; LSCC, laryngeal squamous cell carcinoma; miRNA, microRNA; NPC, nasopharyngeal carcinoma; OL, oral leukoplakia; OPC, oropharyngeal carcinoma; OPSCC, oropharyngeal squamous cell carcinoma; OSCC, oral squamous cell carcinoma*.

**Table 2:** Summary of proteomics in head and neck neoplasms.

**Studied marker**	**Type of tumor**	**No. of patients**	**Sample type**	**Expression**	**Test accuracy indices**			**References**
					**Sens**.	**Spec**.	**Accuracy**	
**CYTOKINES**								
IL-6	OL with dysplasia	20	Saliva	↑	–			(27)
IL-1, IL-6, IL-8, VEGF, and TNF-α	TSCC	18	Saliva	↑	–			(28)
**OTHERS**								
RACK1	OSCC	76	Tissue biopsy	↑	–			(29)
Phosphorylation of ribosomal protein s6 (p-RPS6)	OSCC, oral epithelial dysplasia lesions	68	Tissue biopsy	↑	–			(10)
Adenosine deaminase (ADA)	TSCC	50	Saliva, blood sample	↑	–			(30)
Midkine	HNSCC	103	Blood sample	↑	57.3%	85.3%	–	(31)
NFκB-p50 and IκBα	HNSCC	104	Blood sample	↑	–			(4)
Salivary total protein + soluble CD44 levels (solCD44)	HNSCC (NPC excluded)	102	Salivary rinse	↑	62–79%	88–100%	–	(5)

*HNSCC, head and neck squamous cell carcinoma; NPC, nasopharyngeal carcinoma; OL, oral leukoplakia; OPSCC, oropharyngeal squamous cell carcinoma; OSCC, oral squamous cell carcinoma; TSCC, tongue squamous cell carcinoma*.

**Table 3:** Summary of metabolomics in head and neck neoplasms.

**Studied marker**	**Type of tumor**	**No. of patients**	**Sample type**	**Expression**	**Test accuracy indices**			**References**
					**Sens**.	**Spec**.	**Accuracy**	
**METABOLOMICS**								
Altered energy metabolism (e.g., high glucose, low lactate, low creatine, high creatinine, high choline-creatine ratios etc.)	OSCC	15	Blood sample	↓/↑	–			(32)
Plasma and salivary cortisol levels in the morning	OSCC, OPSCC and OL	68	Blood sample, saliva	↑	–			(33)
A panel of 4 metabolites: choline, betaine, pipecolinic acid, L-carnitine	OSCC	30	Saliva	↓/↑	100%	96.7%	99.7%	(34)
Plasma levels of non-anoic acid, glucose, galactose, and cysteine + cystine	OSCC	48	Blood sample	↓/↑	Non-anoic acid			(35)
					84%	92.9%	–	
					Glucose			
					0.64%	100%	–	
					Galactose			
					92%	92.9%	–	
					Cysteine + cysteine			
					84%	71.4%	–	
Salivary glycine and proline	OCC	79	Saliva	↓	–			(36)

*OCC, oral cavity cancer; OL, oral leukoplakia; OPSCC, oropharyngeal squamous cell carcinoma; OSCC, oral squamous cell carcinoma*.

**Table 4:** Summary of glycomics in head and neck neoplasms.

**Studied marker**	**Type of tumor**	**No. of patients**	**Sample type**	**Expression**	**Test accuracy indices**			**References**
					**Sens**.	**Spec**.	**Accuracy**	
**GLYCOMICS**								
Total sialic acid/total protein (TSA/TP) ratios and α-l-fucosidase	OPC and oral cancer	100	Blood sample, saliva	↑	Serum TSA/TP			(37)
					88.2%	57.2%	–	
					Serum α-l-fucosidase			
					65.9%	82.1%	–	
					Salivary TSA/TP			
					61.2%	44.3%	–	
					Salivary α-l-fucosidase			
					69.4%	48.1%	–	
Sialic acid, total protein, total sugar	OSCC	30	Saliva	↑	–			(38)

*OPC, oropharyngeal cancer; OSCC, oral squamous cell carcinoma*.

**Table 5:** Summary of volatomics in head and neck neoplasms.

**Studied marker**	**Type of tumor**	**No. of patients**	**Sample type (Technique)**	**Expression**	**Test accuracy indices**			**References**
					**Sens**.	**Spec**.	**Accuracy**	
**VOLATOMICS**								
2-butanone, ethanol, 2,3-butanediol, 9-tetradecen-1-ol, octane derivate, cycloheptane derivate, cyclononane derivate	HNEC	11	Breath (SPME-GC-MS)	↑	–			(39)
Ethanol, 2-propenenitrile, undecane	HNSCC	22	Breath (GC-MS, eNose)	↑	(HNSCC vs. HC/benign)			(40)
					77%	90%	83%	
VOC pattern	HNSCC	36	Breath (eNose)	–	HNSCC vs. HC			(41)
					90%	80%	85%	
Ethylhexanol, 4-hydroxybutanoic acid Phenol	PTC	39	Breath (SPME-GC-MS)	↓ ↑	PTC vs. HC			(42)
					100	100	100	
VOC pattern	HNSCC	9	Breath (eNose)	–	–			(43)
Butyric acid, Pentanoic acid, Hexanoic acid, Heptanoic acid	Malignant glottis lesions	6 (T1)	Mucus over lesion (TD-GC-MS)	↑	–			(44)
VOC pattern	HNSCC	52	Breath (eNose)	–	HNSCC vs. LC			(45)
					85%	84%	85%	
Undecane, dodecane, decanal, benzaldehyde, 3,7-dimethylundecane, 4,5-dimethylnonane, 1-octene, hexadecane	PTC	26	Breath (SPME-GC-MS)	↑	PTC vs. HC			(46)
					100	100	–	
Dibutylhydroxytoluene, dimethyl disulphide, decamethylcyclopentasiloxane, methyl ethyl ketone, n-heptane, p-xylene, toluene, I-heptene	OSCC	10	Breath (GC-MS)	↓↑	–			(47)
4-chlorobenzene, methanethiol	HNC	22	Breath (eNose/GC-MS)	–	HNC vs. LC			(48)
					100	100	100	
VOC pattern	HNSCC	100	Breath (eNose)	–	HNSCC vs. colon cancer			(49)
					79%	81%	81%	
					HNSCC vs. bladder cancer			
					80%	86%	84%	
m-cresol, 3-heptanone, benzene, 4-methyl-2-heptanone, acetone, 1-proanol, non-anal, 4-tert-butylphenol, phenol, 3-methyl-2-heptanone, dimethyltrisulfide, 2-hexanone, ethanoic acid, furan, hexanal, 2-methyl-5-(methylthio)furan, heptanal, dimethyldisulfide, 2-methythiophene, tetrahydro-2,2-dimethyl-5-(1-methyl-1-propenyl)furan, 2-methylbutyric acid, styrene, 2-ethylfuran, ethylbenzene, thiophene	HNSCC	53	Urine (SPME-GC-MS)	↑	–			(50)
2-ethyl-5-methylfuran, 3,4-dimethyl-2,5-furanediol, 3,4-dehydro-β-ionone, 2-methylbuanal, linalool, 1,8-cineol, 2-butnaone, α-terpineol, tetrahydro-2,2,5,5-tetramethylfuran, 2-hexenal				↓				
1,4-dichlorobenzene, 1,2-decanediol, 2,5-bis1,1-dimethylethylphenol, E-3-decen-2-ol	HNC	32	Saliva (SPME-GC-MS)	–	1,4-dichlorobenzene (HNC vs. HC)			(51)
					100%	100%		
					1,2-decanediol (HNC vs. HC)			
					100%	80%		
					2,5-bis1,1-dimethylethylphenol (HNC vs. HC)			
					90%	80%		
					E-3-decen-2-ol (HNC vs. HC)			
					80%	80%		
Hydrogen cyanide	HNSCC	23	Breath (SIFT-MS)	↑	Hydrogen cyanide			(52)
					91%	76%		
2-pentanone, undecane, 1,3-butanediol, hexadecanoic acid	OSCC	12	Saliva (GC-MS)	↓ ↑				(53)
					95.8%	94.0%		
VOC pattern	HNSCC	20	Breath (eNose)	–	Detection of recurrence			(54)
					85%	80%	83%	

*eNose, electronic nose; GC, gas chromatography; HC, healthy controls; HNC, head and neck cancer; HNEC, head and neck epidermoid cancer; HNSCC, head and neck squamous cell carcinoma; LC, lung cancer; MS, mass spectrometry; OSCC, oral squamous cell carcinoma; PTC, papillary thyroid carcinoma; PTR, proton transfer reaction; SIFT, selected ion flow tube; SPME, solid phase microextraction*.

**Table 6:** Summary of microbiomics in head and neck neoplasms.

**Studied marker**	**Type of tumor**	**No. of patients**	**Sample type**	**Expression**	**Test accuracy indices**			**References**
					**Sens**.	**Spec**.	**Accuracy**	
**MICROBIOME**								
*Bacillus, Enterococcus, Parvimonas, Slackia*, and *Peptostreptococcus*	OSCC	125	Saliva	↑	–			(55)
*Fusobacterium, Dialister, Peptostreptococcus, Filifactor, Peptococcus, Catonella*, and *Parvimonas*	OSCC	40	Tissue biopsy	↑	–			(56)
*Lactobacillus* and *Streptococcus*	OSCC and OPSCC	19	Oral rinse	↑	–			(57)
*Fusobacterium*	OSCC	4	Tissue biopsy	↑	–			(58)
Firmicutes and Actinobacteria	Oral cancer	15	Tissue biopsy	↓	–			(59)
Oral microbiome panel (*Rothia, Haemophilus, Corynebacterium, Paludibacter, Porphyromonas, Oribacterium*, and *Capnocytophaga*)	OCC and OPC	52	Oral rinse	↑/↓	Oral microbiome panel			(60)
					90%	61%	82%	
*Actinomyces* Parvimonas	HNSCC	121	Tissue biopsy	↓ ↑	–			(61)
*Fusobacterium* *Streptococcus* Microbial diversity	HNSCC	34	Tissue biopsy	↑ ↓	Microbial diversity in HNSCC vs. healthy controls			(62)
					–	–	70%	
*Bacteroides, Pseudomonas, Ruminiclostridium* and *Aggregatibacter*	Throat cancer	32	Saliva	↑	–			(63)
*Streptococcus, Rothia, Porphyromonas, Bulleidia Fusobacterium, Prevotella, Gemella, Granulicatella, Peptostreptococcus, Parvimonas, Peptostreptococcaceae incertae sedis, Catonella, Treponema, Selenomonas, Burkholderia*	LSCC	29	Tissue biopsy	↓ ↑	–			(64)

*HNSCC, head and neck squamous cell carcinoma; LSCC, laryngeal squamous cell carcinoma: OCC, oral cavity cancer; OPC, oropharyngeal cancer; OPSCC, oropharyngeal squamous cell carcinoma; OSCC, oral squamous cell carcinoma*.

**Table 7:** Summary of radiomics in head and neck neoplasms.

**Imaging technique**	**Type of tumor**	**No. of patients**	**Predictive model**	**Test accuracy indices**			**References**
				**Sens**.	**Spec**.	**Accuracy**	
CT scan	Thyroid nodule	103	Tumor type cytokeratin 19	93%	73%	87%	(65)
			Tumor type galectin 3	87%	76%	85%	
			Tumor type thyroperoxidase	86%	75%	84%	
Ultrasound	Thyroid nodule	137 in training set 95 in validation set	Malignant nodules	–	–	93%	(66)
CT scan	HNSCC	136 in training set 95 in validation set	Multivariate HNC signature to predict tumor stage	–	–	80%	(67)
MRI	HNSCC	85 in training set 42 in validation set	Combined T2W and ceT1W radiomic features to predict tumor stage	84%	70%	79%	(68)
			ceT1W radiomic features to predict tumor stage	80%	67%	75%	

*HNSCC, head and neck squamous cell carcinoma; HNC, head and neck cancer*.

**Table 8:** Summary of other biomarkers for head and neck neoplasms.

**Studied marker**	**Type of tumor**	**No. of patients**	**Sample type**	**Expression**	**Test accuracy indices**			**References**
					**Sens**.	**Spec**.	**Accuracy**	
Higher mean platelet volume (MPV)	PTC	66	Blood sample	↑	60%	80%	73%	(69)

*PTC, papillary thyroid carcinoma*.

### Genomics

In the genomic approach, various methods were used to analyze genetic aberrations such as DNA sequencing, single nucleotide polymorphism analysis and hybridization techniques. Changes in gene expression are considered as a potential biomarker. A benefit of this approach is that it might also reveal the disease's underlying cause. On the other hand, there are also some demerits. Cancer is a condition in which many genes interact. Therefore, a single biomarker is probably insufficient to diagnose head and neck cancer and a biomarker panel of genes is recommended. It might also be unclear which post-transcriptional regulatory processes are involved (70). Table 1 and Supplementary Table 1 summarize the biomarkers involving genomics.

#### Promoter Hypermethylation

Promoter hypermethylation refers to the epigenetic process of abnormal methylation of CpG-islands in the promoter region. The promoter regions of genes initiate gene transcription and are usually not methylated (12). These alterations are associated with gene silencing in cancer (14). Methylation is suggested to be an early event in carcinogenesis (3, 14), which makes it an interesting candidate as a biomarker. In this manner, early diagnosis is potentially achieved and consequently leads to a better prognosis. Different papers focused on promoter hypermethylation and show that specific genes of patients with head and neck neoplasms have a higher prevalence of methylation in comparison to a healthy control group (3, 6, 11, 12). Sushma et al. suggest a promoter hypermethylation panel for oral squamous cell carcinoma (OSCC) consisting of the following genes: PTEN and p16 (12), which can be detected by a tissue biopsy. Guerrero et al. also suggest a promoter hypermethylation panel for detection of OSCC with the genes HOXA9 and NID2, identified via tissue biopsy or salivary rinses (3). However, despite a high specificity of promoter hypermethylation as a biomarker, its sensitivity was low. Using a panel of genes increased the sensitivity but came at the cost of a lowered specificity. Moreover, in these studies, the included population was small and/or consisted of high-risk patients. Further research would thus be necessary to explore this potential marker.

#### Epstein-Barr Virus DNA Load

Nasopharyngeal carcinoma is closely associated with a latent Epstein-Barr virus (EBV) infection (13, 71). The EBV DNA load has biomarker potential [sensitivity of 95% (95% CI, 91–98%), specificity of 98% (95% CI, 96–99%) (14)]. The serum concentration correlates to the tumor burden, resulting in a low specificity in early tumor stages but pointing toward the potential of a good prognostic biomarker (high concentrations indicating a greater tumor mass and thus negative prognosis). A combination of the EBV DNA load with a marker for early detection would increase the sensitivity for an early stage detection of nasopharyngeal carcinoma. Yang et al. therefore suggested to combine EBV DNA load with a panel of hypermethylation markers for detection of nasopharyngeal carcinoma (13), since methylation is an early event in carcinogenesis (3, 13).

Several antibodies to EBV were also investigated as a biomarker for the diagnosis of nasopharyngeal carcinoma, for example anti-EBV capsid antigen IgA (IgA-VCA) (71). This is further discussed under the paragraph “proteomics.” Epstein-Barr virus DNA load [95% (95% confidence interval, 91–98%)] and IgA-VCA [sensitivity of 81% (95% CI, 73–87%)] are two of the most sensitive biomarkers found in the peripheral blood of nasopharyngeal carcinoma patients. When combined, a sensitivity of 99% was obtained. The specificity of EBV DNA and IgA-VCA was 98% (96–99%) and 96% (91–98%), respectively. On top of this, because of their different production mechanism, the combination of both markers could minimize false positive cases (14). With its high sensitivity and specificity, this biomarker panel of EBV DNA and IgA-VCA seems very promising in the diagnosis of nasopharyngeal carcinoma. Further prospective validation studies in independent cohorts are needed for confirmation and determination of its position in the diagnostic landscape.

#### Human Papillomavirus DNA Load

As already mentioned, human papillomavirus (HPV) and Epstein-Barr virus (EBV) are identified viral risk factors for head and neck neoplasms (72). Since HPV-16 accounts for >90% of HPV-DNA positive head and neck squamous cell carcinomas (HNSCC), it is regarded as the predominant HPV type in these specific malignancies (73). As a result, this is currently the only HPV type that has been studied in HNSCC.

The oral HPV-16 prevalence in healthy individuals is ~1%, suggesting HPV sequences could be used as a biomarker to detect the associated neoplasms (73). An important association has been demonstrated between HPV and oropharyngeal squamous cell carcinomas (OPSCC) on the one hand and some non-oropharyngeal head and neck squamous cell carcinomas on the other hand such as cancers of the oropharynx, larynx, and hypopharynx (73). In general, the survival rate was found to be higher for tumors that were HPV-positive compared to the neoplasms that were HPV-negative (24), stressing the prognostic property. Besides this classification, tumors can also be divided in HPV-driven and non-HPV-driven cancer. HPV-driven malignancies are, amongst other things, characterized by at least one HPV genome copy per tumor cell, as opposed to non-HPV-driven malignancies which express only low copy numbers of HPV DNA (23). Holzinger et al. state that HPV-driven OPSCC are classified as a distinct tumor entity and have specific characteristics, of which a better patient survival is of the biggest clinical importance (23). In contrast, patient survival in HPV-positive but non-HPV-driven OPSCC is similar to that of patients with HPV-negative cancer (23). Kreimer et al. showed, in a small subset of tumor specimens (*n* = 9), that the sensitivity of antibodies to HPV-16 oncoprotein E6 (HPV-16 E6) for detection of HPV-driven OPC in blood, was exceptionally high (estimated at 100%, 95% CI = 47.8–100%) with a specificity that was also in this range (estimated at 100%, 95% CI = 39.8–100%) (22). Holzinger et al. supported this statement and showed that HPV-16 E6 seropositivity had a very high sensitivity (96%) and specificity (98%) to diagnose HPV-driven OPSCC. In contrast, the sensitivity for diagnosis of HNSCC excluding oropharyngeal carcinoma, was much lower (50%, 95% CI = 19–81%) despite the very high specificity (100%, 95% CI = 96–100%) (23).

Regarding sampling methods, HPV DNA load, and HPV antibodies can be detected in plasma as well as in saliva (23, 24, 26, 72). Wang et al. demonstrated that HPV DNA could be detected in the plasma of 86% of the patients, compared to only 40% of the saliva of these same patients, indicating that plasma would be more informative to diagnose HPV-associated tumors despite the need for invasive sampling (24). Indeed, Kreimer et al. found that in OPSCC, the sensitivity for HPV-16 DNA detection in saliva was found to be between 45 and 82% compared to a sensitivity of ≥90% when HPV-16 antibodies were detected in serum (73).

These HPV-related markers do have their limitations as well. First of all, Kreimer et al. indicate that HPV-P16 E6 seronegative individuals have a low risk of developing HPV-driven OPC but that a screening test for these antibodies in the general population, would still lead to a significant amount of false-positive results. Thus, identifying the population at risk for OPSCC would improve the positive predictive value of this biomarker (73). Another remark, which is also noticed by Wang et al. and Kreimer et al., is that the published studies consisted of small study groups and studies with greater statistical power are required to determine the possibility of using these HPV related markers in detecting not only neoplasms of the oropharynx, but also the larynx and hypopharynx (24, 73).

#### MicroRNA

In the last two decades, altered microRNA expression were studied in different solid tumors and hematological malignancies. These microRNAs are single-stranded non-coding RNAs of 17–25 nucleotides that circulate in cell-free body fluids like blood plasma, serum, saliva, and urine. They can bind to a complementary site in 3′-untranslated regions of the messenger RNA (mRNA), thereby negatively regulating the gene expression via mRNA degradation or translational inhibition. Some microRNAs are upregulated in cancers and are regarded as oncogenes. Others are downregulated and are thus presumed to be tumor suppressor genes. Tumor-derived microRNAs are also released into the blood and might thus be potential early cancer detection markers. Furthermore, these microRNA profiles can be retrieved in a minimally invasive way and they are very stable, up to 28 days, in serum and plasma when stored at −20°C or below (7, 15). There is a plethora of papers that studied microRNAs, resulting in a large number of microRNAs that have been identified. MicroRNAs have been studied as a marker for oral cancer (15, 16) and larynx cancer (7, 17, 74). Promising results have been observed for a combination of miR-657 and miR-1287 as a marker for larynx carcinoma with a sensitivity of 86% and a specificity of 100% (74). Nevertheless, the result of this study needs to be interpreted with caution, since it was not yet validated in independent cohorts. The same counts for all other microRNAs that were under investigation in the aforementioned studies.

#### Interferons-Related Genes

The interferons belong to the family of multifunctional cytokines. These cytokines are produced by host cells in response to microbial infections and tumor cells. When secreted, they initiate a cascade through JAK/STAT signaling (Janus Kinases/Signal Transducer and Activator of Transcription) on their turn resulting in interferon-stimulated gene upregulation. To date, more than 400 interferon-stimulated genes have been reported (18, 19). The first one to be recognized was ISG15 and has been described in many tumor biopsies from several cancers including oral squamous cell carcinoma (19). Due to this fact, it might not be a very specific marker for head and neck neoplasms. Another candidate gene is the interferon-inducible transmembrane protein 1 gene (18). The exact mechanism resulting in overexpression of interferon-stimulated genes in tumor cells is not yet clarified. Current ongoing studies aim to identify interferon-stimulated genes in diverse tumors including oral squamous cell carcinoma (18).

### Proteomics

A promising approach to identify biomarkers is the study of cell proteins, called proteomics (29, 70). Protein markers like carcinoembryonic antigen (CEA), prostate specific antigen (PSA), alpha-fetoprotein (AFP), and cancer antigen 125 (CA-125) already earned their place in the diagnosis or progression of several cancer types (75). In head and neck squamous cell carcinoma, the following markers have been studied: NFκB-p50, IκB (4), and the growth factor midkine (31) by blood sampling, and total salivary protein combined with soluble CD44 levels (solCD44) in saliva (5). In oral squamous cell carcinoma, a link has been shown with the protein receptor for activated C kinase 1 (RACK1) (29). Furthermore, there is also a place for detection of cytokines in saliva, for example IL-6 in oral leukoplakia or IL-1, IL-6, IL-8, VEGF, and TNF-α in tongue squamous cell carcinoma (27, 28). Although some proteins were put forth as a biomarker for oral squamous cell carcinoma, several problems limit their clinical utility. First, there is a substantial heterogeneity of biomarker expression. Second, some proteins are also expressed in other pathologic situations such as inflammatory conditions resulting in a low specificity. Third, different experimental protocols were used which might explain the discrepancy between the identified biomarkers (70). Because of the heterogeneity of tumor markers in different patients, a combination of proteins might again be a better approach to increase their utility. Although protein markers seem very promising, there has not yet been identified a single or a combination of biomarkers to be effective for clinical use.

Several studies have suggested that autoantibodies that target specific tumor-associated antigens, could possibly be detected years before the tumor can be discovered through incidence screening or radiography (76). During early carcinogenesis, our immune system tries to remove precancerous lesions by generating an immune response to specific tumor-associated antigens (76, 77), which makes them suitable for early detection of cancer lesions. Besides, autoantibodies are found in serum, which is favorable for screening. These individual autoantibodies, however, lack the sensitivity and specificity required for cancer screening (14, 76).

First of all, some specific tumor-associated antigens can arise in different types of cancer (e.g., p53) and some of them are also present in diseases other than cancer, especially autoimmune diseases (e.g., rheumatoid arthritis, diabetes mellitus type 1, systemic lupus erythematosus…). They can also be detected in non-cancer individuals, and because of the heterogenic nature of cancer, a single autoantibody can only be found in 10–30% of patients with the same type of cancer. A panel of specific tumor-associated antigens might hence be the key to raise sensitivity and specificity (76). As mentioned before, there were several antibodies to Epstein-Barr virus (EBV) investigated as a biomarker for the diagnosis of nasopharyngeal carcinoma, anti-EBV capsid antigen IgA (IgA-VCA), and anti-EA IgG individually, appeared to have the greatest potential (71). A combination of IgA-VCA and Epstein-Barr virus DNA load could minimize false positive cases [(14); Table 2].

### Metabolomics

The term metabolome refers to the identification and quantification of all the small molecule metabolites (<1 kDa) in tissue or biofluids produced during cell metabolism (36). It is directly linked to cell physiology and thus the result of both physiological and pathological metabolic processes. This explains the current use of metabolomics for discovery of novel biomarkers for cancer diagnosis (36). However, there are some drawbacks to its use. Because of the high complexity of the metabolome, interpretation of data is difficult, urging the use of deep learning and data mining techniques. There is also a big difference in concentrations ranging for nanomolar to millimolar. Diet, sex, age, drugs, environment, and lifestyle might interfere with the metabolite concentration (70). There are some papers describing metabolome-related biomarkers in oral cancer. Bernabe et al. found that the plasma and salivary cortisol levels were significantly higher in patients with oral squamous cell carcinoma in comparison with healthy controls and patients with oral leukoplakia (33). Large validation studies remain indispensable to confirm the value of metabolites in the diagnosis of head and neck neoplasms. To date, research on metabolites as a potential biomarker is still in the discovery phase.

### Glycomics

Compared to genomics and proteomics, there is few research on glycomics. This approach focuses on the modifications of glycoconjugates related to cancer. Glycolipids and glycoproteins are glycoconjugates and are important constituents of the cell membrane. Glycosylation is important in the process of protein modification and its action relies on the function of glycosyltransferases and glycosidases in various tissues and cells. Glycoconjugates are released into the circulation because of continuously shedding and/or secretion by cancer cells or the increased cell turnover and could thus be detected in body fluids to be used as tumor markers. Especially glycoconjugates in oral cancer, which are in direct contact with saliva, seem to be promising. The major types of glycosylation are sialylation and fucosylation, which terminally modify proteins that are important in the vital biological functions. There have been reported significantly elevated levels of sialic acid, α-l-fucosidase, and total protein in oral cancer patients and there is also a link with oral cancer progression. However, there is need for further research to determine the role of these biomarkers in oral cancer development (37, 38).

### Volatomics

Volatomics recently emerged as new research field for early disease diagnosis. This encompasses volatile organic compounds (VOCs) in nano- to picomolar concentrations, which are the gaseous end products from endogenous metabolic changes, digestion, microbiome, inflammation, and oxidative stress. VOCs can be detected in breath, urine, feces, blood, saliva, skin, and sweat, and hence, serve as attractive biomarkers, as it is completely non-invasive, relatively cheap, and provides rapid results (78). VOCs have already shown clinical potential as biomarkers for lung (79), gastric (80), breast (81), prostate (82), and mesothelioma cancer (83), and since carcinogenesis is related to inflammation and metabolic changes, VOCs could also have added value as diagnostic biomarkers for head and neck cancer (Table 5).

The study of VOCs in exhaled breath (breathomics) is potentially the most important since the sample is unlimitedly present and sampling causes no side effects for the patient. Using gas chromatography-mass spectrometry (GC-MS), García et al. found an increase of 2-butanone, ethanol, 2,3-butanediol, 9-tetradecen-1-ol, and octane, cycloheptane, and cyclononane derivates in the breath of head and neck cancer patients compared to healthy controls [(39); Table 5]. The increase in ethanol was also found in head and neck squamous cell carcinoma (HNSCC) patients by Gruber et al., next to 2-propenenitrile and undecane (40), which discriminated patients from healthy controls and even patients with benign conditions with 77% sensitivity and 90% specificity. Hydrogen cyanide was found to be increased in the breath of HNSCC patients compared to controls using SIFT-MS and allowed discrimination with 91% sensitivity and 76% specificity (52).

The VOCs 4-chlorobenzene and methanethiol discriminated HNSCC patients from lung cancer patients with 100% accuracy (48), showing potential to use breath analysis for differential diagnosis, albeit with low study participants and a risk of overfitting the differentiating models.

Next to spectrometric analysis, VOCs can be detected by sensor technology [electronic noses (eNoses)] that recognizes the bulk of VOCs as a breath print, but without identifying individual VOCs. Using eNoses, HNSCC patients could be differentiated from controls with sensitivities and specificities ranging between 77–90% and 80–90%, respectively, underlining the difference in breath print and their use as diagnostic tool (40, 41, 43). Also, eNoses have shown to be promising for differential diagnosis, in which the breathprint of HNSCC patients was different from those with lung cancer, colon cancer and bladder cancer with, respectively, 85, 79, and 80% sensitivity and 84, 81, and 86% specificity (45, 49, 54).

Two studies discriminated patients with papillary thyroid carcinoma (PTC) from healthy controls with both 100% sensitivity and specificity (42). Although one based this discrimination on an increase of ethyl hexanol, 4-hydroxybutanoic acid, and a decrease in phenol (42), the other did not report changes in these compounds (46).

Differences in dibutylhydroxytoluene, dimethyl disulphide, decamethylcyclopentasiloxane, methyl ethyl ketone, n-heptane, p-xylene, toluene, I-heptene were found in breath between patients with oral squamous cell carcinoma and controls (47). However, based upon VOCs from saliva in these patients, an increase in 1,3-butanediol and hexadecenoic acid and a decrease in 2-pentanone and undecane allowed their discrimination from healthy controls with 96% sensitivity and 95% specificity (53). This decrease of undecane is in contrast to an increase found in breath in HNSCC (40) and patients with PTC (46). Furthermore, saliva analysis of the single VOCs 1,4-dichlorobenzene, 1,2-decanediol, 2,5-bis1,1-dimethylethylphenol, and E-3-decen-2-ol allowed to discriminate HNSCC patients from controls with a sensitivity and specificity between 80–100% and 80–100%, respectively (51), again being different to VOCs found in exhaled breath and urine (50).

Lastly, analysis of VOCs from the mucus of 6 patients with malignant glottic lesions found butyric acid, pentanoic acid, hexanoic acid, and heptanoic acid to be different compared to controls (44).

Carcinogenesis is related to an altered metabolism, upregulated aerobic glycolysis (known as the Warburg effect) and induces oxidative stress (84, 85). This liberates highly reactive oxygen species (ROS) which induce lipid peroxidation of (poly)unsaturated omega-3 and omega-6 fatty acids (PUFA) in the cellular membranes, mainly generating alkanes and aldehydes as end products (86). Considering the high number of hydrocarbons detected in several matrices, this plays a major role in HNC and therefore, are biomarkers of interest. Aldehydes are furthermore generated *in vivo* in signal transduction, genetic regulation and cellular proliferation. In cancer, an increase in aldehyde dehydrogenase is seen as malignant cells proliferate. This causes aldehyde oxidation, resulting in an increased aldehyde concentration in blood and breath, which is reflected by the large number of aldehydes found in these matrices. However, longer chain aldehydes are potentially by-products of digestion and their origin needs to be explored. Also, several organic (carboxylic) acids have been found, which are the main products of proteolysis. Alcohols have 2 major pathways to be induced *in vivo*: by ingestion or as product from the hydrocarbon metabolism by cytochrome P450 enzymes and the alcohol dehydrogenase activity (86). Alcohols, next to carboxylic acids, are products of hydrolysis of esters. The cytochrome P450 enzymes hydroxylate lipid peroxidation biomarkers to produce alcohols, which are found by several studies. Special attention can be given to phenol: although phenol may be derived from benzene metabolism, it is most likely to be from exogenous origin, since it is a by-product of the sampling materials used.

Taken together, it seems that not one VOC is able to accurately discriminate between several types of head and neck cancer types and controls. Hence, the combination of several biomarkers into panels will therefore be key in future research as stressed by the success of eNoses that react to the bulk of VOCs in the breath and the success of biomarker panels. However, the discordance in VOCs can be explained due to a difference in technology, sampling and by the difference in concentration range. Furthermore, as with metabolites, the volatilome delivers high throughput data, complicating the interpretation of the data, and urging the use of deep learning and data mining techniques. Next to this, also diet, sex, age, drugs, environment, and lifestyle, and the microbiome can interfere with the VOC concentration and induce changes. Hence, the external influence may not be underestimated. In several trials, ethanol, and 2-propenenitrile have been identified as possible biomarkers. However, these can be linked to alcohol abuse and smoking, which are both risk factors for HNSCC, and could therefore have biased the results if not corrected for this. Furthermore, the finding that hydrogen cyanide can serve as biomarker raises concerns about the origin of this VOC and its correlation with HNSCC since hydrogen cyanide is known to be released by the microbiome and could result from exogenous exposure to exhausted fumes and cigarette smoke. Despite this, it can also be a by-product of cellular respiration. Hence, for multiple VOCs, their origin and its role as diagnostic marker remains to be determined.

### Microbiomics

The human body is colonized by numerous microbes that include viruses, bacteria and microbial eukaryotes. Studying these microbial communities is referred to as “microbiomics” (64). The microbiome maintains homeostasis and has a dynamic association with the human host (61, 62). When dysbiosis or ecological imbalance arises, processes leading to a diseased state develop (62). Some studies that have already been published, showed an association between microbiome variations and cancer. These studies have demonstrated that the mucosal layers of the mouth, throat, stomach, and intestines are colonized by commensal bacteria, which play an important part in normal human health and can therefore also play a role in the development of malignancies. For example, *Helicobacter pylori* infection can induce gastric cancer through gastric dysbiosis (63).

To date, few has been published about microbiomics as a (diagnostic) biomarker in head and neck cancer. Most studies focus on the oral microbiome, which can be used in the detection of oral cancer, especially oral squamous cell carcinoma (OSCC) (55). There are many bacterial species in the oral cavity involved in the genesis of oral cancer which can be explained through inflammation-induced DNA damage of epithelial cells caused by endotoxins secreted by these micro-organisms (55). The link between microorganisms and head and neck squamous cell carcinoma (HNSCC) is not yet adequately studied (57). There are some studies that have detected oral microbial alternations in consumers of alcohol, tobacco and betel nut. The association between these known oral cancer risk factors and microbial alterations should be taken into account (55, 61). It could be possible that the microbiome helps change an environmental exposure into a carcinogenic effect (61). Lee et al. studied microbial differences between oral cancer patients, patients with epithelial precursor lesions and healthy controls (55). They found a significant abundance of 5 genera in the salivary microbiome (*Bacillus, Enterococcus, Parvimonas, Slackia*, and *Peptostreptococcus*) of cancer patients when compared to patients with epithelial precursor lesions. These changes in the composition of the microbiome could thus be a potential biomarker for monitoring the transformation of oral precursor lesions into oral cancer (55). The use of a microbiome panel (*Rothia, Haemophilus, Corynebacterium, Paludibacter, Porphyromonas, Oribacterium*, and *Capnocytophaga*) could detect oral cavity cancer and oropharyngeal cancer by oral rinse with an accuracy of 82% (60).

The association between variations in the human microbiome and throat cancer has also been studied. Wang et al. studied microbial markers in the saliva of patients with throat cancer (hypopharyngeal carcinoma and laryngeal carcinoma) and in the saliva of patients with vocal cord polyps and healthy controls (63). They revealed a significant difference in the microbiome of throat cancer patients vs. patients with vocal cord polyps and healthy controls. The following genera were found to be associated with throat cancer: *Bacteroides, Pseudomonas, Ruminiclostridium*, and *Aggregatibacter*. Additionally, they observed a significant reduction in microbial diversity in throat cancer patients (63). This reduction in diversity of the microbiome is also found in other studies concerning oral cancer (57, 60) and head and neck squamous cell carcinoma (HNSCC) (62). Zhao et al. on the other hand, observed a greater bacterial diversity in OSCC tissue when compared to healthy tissue (56).

Table 7 shows potential microbial biomarkers for head and neck cancer. Few studies have shown that the expression of *Fusobacterium* is elevated in the tumor tissue of patients with OSCC (56, 58) and HNSCC (62).

Although studies of bacteria and their role in carcinogenesis of colorectal cancer are increasing rapidly, the association between microbiome and head and neck cancer has not been substantially studied. The published studies do not specifically focus on early diagnosis of head and neck neoplasms and have only identified some potential biomarkers based on difference in expression between head and neck cancer populations and (healthy) control populations. These studies also use different sequencing technologies to measure the abundance of microbiota. There is need for further, more standardized research before microbial variations can be considered a diagnostic biomarker for head and neck neoplasms.

### Radiomics

Radiomics comprises the high-throughput mining of advanced quantitative features to objectively and quantitatively describe tumor phenotypes. It makes use of standard of care radiologic images which are subject to advanced mathematical algorithms to detect tumor characteristics that might be missed by the radiologist's eye. This method relies on big data and supports the clinical decision to diagnose, prognose, and predict -amongst others- cancer (87). Yip and Aerts extensively reviewed the applications and limitations of this new -omics approach (88). We kindly refer to their paper for detailed information.

In head and neck cancer, radiomics has also entered the scene. Here, we focus on papers that aimed to diagnose HNC based on radiomics features. In patients with a thyroid nodule, a radiomics score (calculated from ultrasound images) was evaluated against the standard method used for the diagnosis of thyroid nodules as set by the American College of radiology, the TI-RADS score. The radiomics score was able to discriminate malignant from benign nodules with an accuracy of 93% [95% CI 88.4–97.7%]. The radiomics score performed better compared to the TI-RADS if scored by junior radiologists (66). In a similar population, a radiomics predictive model was constructed based on computer tomography (CT) images which was able to predict the immunohistochemical characteristics of suspected thyroid nodules [cytokeratin 19 (AUC 0.87, sensitivity 93%, and specificity 73%), galectin 3 (AUC 0.85, sensitivity 87%, and specificity 76%), and thyroperoxidase (AUC 0.84, sensitivity 86%, and specificity 75%)] (65). It seems clear that radiomics will become a meritorious player in the diagnostic landscape of thyroid cancer. Given the high prevalence of -largely benign- thyroid nodules, a good biomarker to discriminate benign from malignant nodules is indispensable. From the papers published to date, radiomics seems promising as a biomarker in this field.

Parmar et al. were able to identify 10 radiomic clusters in a dataset of CT images from 136 patients with HNSCC that were significantly associated with tumor stage (67). In addition, they created an HNC signature based on multivariate analysis that was highly predictive for tumor stage (AUC = 0.80). In a similar population consisting of 127 HNSCC patients, Ren et al. used magnetic resonance imaging (MRI) axial fact-suppressed T2-weighted (T2W) and contrast-enhanced T1-weighted (ceT1W) images to identify a radiomics signature for preoperative staging (I-II vs. III-IV). The radiomics signature based on ceT1W images (AUC 0.853) performed best in discriminating stage I-II from stage III-IV followed by combined T2W and ceT1W images (AUC 0.849) (68). In the training cohort, radiomics performed better than visual assessment by an experienced radiologist, however, this was no longer the case in the testing cohort. In a recent paper of Huang et al., the radiomics' potential to identify treatment-relevant subtypes of HNSCC was tested on pretreatment CT scans in a cohort of 113 patients. Moderate AUC's varying from 0.71 to 0.79 were observed in the prediction of HPV positivity, three DNA methylation subtypes and a mutation of NSD1 in these patients (89). Radiomics might thus be of additional value to define subtypes in a non-invasive manner. However, several tumors will be misclassified based on radiomics alone, making the diagnostic capacity underperforming.

Besides tumor diagnosis, radiomics is also subject of investigation in predictive (90–92) and prognostic models (67, 93–96), to evaluate local tumor control (97–99), and HPV status (100, 101).

A challenge in radiomics remains the fact that the extracted feature quality is affected by tumor segmentation methods used to define regions over which to calculate features. Consistent radiomics analysis across multiple institutions that use different segmentation algorithms are not obvious. This is particularly the case for Positron Emission Tomography (PET), where a limited resolution, a high noise component related to the limited stochastic nature of the raw data, and the wide variety of reconstruction options might confound quantitative feature metrics (102). Standardized scanner protocols and image reconstruction harmonization are thus of tremendous importance to make the transfer of radiomics features possible between institutions. Several papers already tried to identify the pitfalls of radiomics and the relevant features that delay interchangeability to make sure that radiomics becomes a full-fledged biomarker in future cancer diagnosis (103).

### Others

Mean platelet volume (MPV) was found to be significantly higher in PTC patients when compared to benign goiter patients and health controls (8.05, 7.57, and 7.36 fl, respectively; *p* = 000.1; see Table 8). Moreover, MPV significantly decreased when these patients were surgically treated [8.05 vs. 7.60 fl; *p* = 0.005; (69)]. The quality of this study however was suboptimal. The study was retrospective and had a relatively small sample size [*n* = 66; (69)].

## Discussion

In this review, we assessed a large number of potential biomarkers for the diagnosis of head and neck neoplasms and included tables providing a detailed overview of the state-of-the-art investigated biomarkers. All proposed biomarkers have their advantages and restrictions. Many biomarkers lack the sensitivity and/or specificity that is required for utilization in the clinical practice. Therefore, individual biomarkers are frequently combined into biomarker panels to increase these diagnostic values.

Furthermore, many studies had a relatively small sample size and therefore lacked statistical power. Additionally, a great amount of these studies was retrospective. Larger, prospective studies should thus be performed in the future. Also, other elements should be kept in mind when planning future biomarker research. For example, a considerable part of the studies that were reviewed included healthy controls as control population. However, one should recognize that biomarkers in the clinical practice would be used in patients at risk for certain types of head and neck neoplasms. Future studies should thus consider including appropriate patients at risk and/or patients known with a premalignant lesion when composing their control population. Most of the described markers have been studied for one specific type of head and neck neoplasm and can therefore not be extrapolated to head and neck neoplasms in general. The diagnostic biomarkers that were reviewed, were frequently studied in patients from one specific geographical location. As a consequence, the biomarker might be influenced by race, genetics, lifestyle, and carcinogenic exposure (104). India is a high-risk region for the development of oral squamous cell carcinoma (12, 18, 19, 38). Hence, a biomarker that has been evaluated in an Indian community might be applicable only to high-risk regions. The study groups should thus also investigate this marker in another population that is less at risk for the occurrence of oral squamous cell carcinoma and study its applicability worldwide. Also, other variables such as race, eating habits and environmental exposure should be taken into account. In this manner, when reviewing the literature, we noticed a large amount of variability when studying biomarkers for head and neck cancer. Standardized research would therefore be a necessity when considering future studies concerning these tumors.

The sampling method of the biomarkers and the analysis of the specimen also play a determining role in the utilization of the marker in the clinical practice. For example, saliva and exhaled breath present an attractive non-invasive alternative to tissue or serum testing. Serum is easily accessible, relatively cheap, and can be used in a minimally invasive way, but saliva and breath offer some other advantages. A non-health practitioner can easily collect these samples in a non-invasive way, they are easy to store, the sample cost is relatively low and repeated (unlimited) samples can easily be acquired because it is a comfortable sampling method for patients (34, 37).

To our opinion, microRNA, gene hypermethylation, HPV-related markers, and a panel of proteins seem to be the biomarkers with the most promising potential of becoming a diagnostic biomarker for head and neck neoplasms based on the reported sensitivity, specificity, positive, and negative predictive values that can be obtained. In certain tumor types, such as thyroid cancer, also radiomics might become of importance in the diagnostic landscape and replace invasive needle biopsies. Hereto, radiomic signatures need to be identified that are able to discriminate benign from malignant lesions. A challenge here is to make the radiomic signatures interchangeable between institutes. Biomarkers studying the metabolome, glycome, volatilome, and microbiome still need to be thoroughly investigated before they can be considered as biomarkers.

## Conclusion

Most of the head and neck tumors are diagnosed in an advanced stage. Hence, besides advancement in treatment of head and neck neoplasms, early detection of these tumors could play a significant role in improving the prognosis of these patients. With this in mind, a lot of research has already been done on clinically applicable single biomarkers or a panel of biomarkers, for early detection of head and neck tumors. We reviewed over 50 markers, all with their advantages and limitations. To date, a biomarker to diagnose head and neck neoplasms useful for clinical practice, has not yet been identified nor validated. Therefore, further research of biomarkers to diagnose head and neck neoplasms in an early stage is still needed.

## Author Contributions

HK, SS, and MG collected, analyzed, and interpreted the data after which they drafted the article. This excludes the part concerning volatomics, which should be accredited to KL and the part concerning radiomics, which should be accredited to KJL. KJL, KL, JM, OV, BD, and PS did a critical revision of the article and gave their final approval of the version to be published. All authors contributed to the article and approved the submitted version.

## Conflict of Interest

The authors declare that the research was conducted in the absence of any commercial or financial relationships that could be construed as a potential conflict of interest.
