# Supplementary material for: A Literature Review of the Potential Diagnostic Biomarkers of Head and Neck Neoplasms
Source: Front Oncol. 2020 Jun 26;10:1020. doi: 10.3389/fonc.2020.01020 (PMC7332560; doi:10.3389/fonc.2020.01020)
Supplement: Supplementary file 1 [file Data_Sheet_1.docx]

# **Supplementary tables**

| **Supplementary table 1: diagnostic biomarkers for head and neck neoplasms - GENOMICS** | | | | | | |
| --- | --- | --- | --- | --- | --- | --- |
| **TYPE OF TUMOUR** | **POTENTIAL BIOMARKER** | **ADVANTAGES** | **RESTRICTIONS** | | **STUDY POPULATION** | **REFERENCE** |
| **(PROMOTOR) HYPERMETHYLATION** | | | | | | |
| OSCC | **A promotor hypermethylation panel (HOXA9 and NID2)** | - Promotor hypermethylation is known to be an early event in carcinogenesis. - Combination of individual genes with 94% sensitivity and 97% specificity in tissue samples, 75% sensitivity and 53% specificity in saliva samples. - HOXA9 alone (85% sensitivity and 97% specificity). - NID2 alone (87% sensitivity and 95% specificity)*.* | - A very indistinct description of their research population was given. - This panel should be studied in larger cohorts. | | - Patients from hospitals in Baltimore (USA, n=143), and Madrid (Spain, n=36) were included. - No further details were given. | (Guerrero-Preston et al., 2011) |
| OSCC, OPSCC | **Methylation of cg01009664 of the thyrotropin-releasing hormone (TRH) gene** | - A significant difference in methylation percentage of the TRH-gene was demonstrated between cancerous and healthy cells (63% ± 19.81% versus 7% ± 3.43%). - Real-time polymerase chain reaction (PCR) used on the obtained epithelial cells, showed a significantly higher DNA methylation level in oral swab and oral rinse samples from OSCC and OPSCC patients compared to healthy controls (p < 0.001). - Promotor hypermethylation is known to be an early event in carcinogenesis. - It is an easily accessible biomarker (oral rinse, oral swab). | - The study consists of a rather small sample group. - The researchers did not find a difference between the clinical stage or histological grade and TRH methylation level. | | - In this study methylation microarray data were used from the Gene Expression Omnibus (GEO) repository of NCBI using bioinformatics. - 18 samples of mucosal tissue to validate the obtained data using pyrosequencing. - OSCC patients (n=9) of whom 5 male and 4 female. - Healthy controls (n=9) of whom 4 male and 5 female. - Epithelial cells from 2 cohorts to evaluate TRH-methylation levels using real-time PCR. - Discovery set: oral rinse and oral swab from OSCC patients (n=23), and oral rinse from healthy controls (n=33). - Validation set: oral rinse from OSCC (n=42) and OPSCC (n=24) patients, and oral rinse from healthy controls (n=54). | (Puttipanyalears et al., 2018) |
| OSCC | **Promotor hypermethylation panel (PTEN and p16)** | - Methylation of PTEN and P16 was significantly higher in OSCC tissue compared to the healthy tissue samples. - Promotor hypermethylation is known to be an early event in carcinogenesis. | - The study consists of a rather small sample group. - Only tested in high-risk area for OSCC. | | - Tissue samples from 50 patients with OSCC containing tumour tissue and surrounding healthy tissue. - The patients were all originating from South India. | (Sushma et al., 2016) |
| NPC | **EBV DNA load + hypermethylation panel (RASSF1A, WIF1, DAPK1 and RARβ2)** | - Methylation is an early event in carcinogenesis, what makes this an ideal biomarker to combine with EBV DNA load for detection of NPC, which has a low specificity in early tumour stages. - The serum concentration of EBV DNA load correlates to tumour burden, which might make it a good candidate for a prognostic biomarker. | - Until today, this biomarker has only been studied in patients with NPC. Other tumour groups should thus be tested in the future. - The predictive value of EBV DNA load alone is not high enough to perform well in high risk populations. - The reproducibility of quantitative plasma EBV DNA does not perform well. - The serum concentration of EBV DNA load correlates to tumour burden, indicating a low specificity in early tumour stages. | | - NPC patients (n= 252), of whom 72% were male, mean age of 52y. - High-risk patients (n=43), of whom 42% were male, mean age of 49y. - Non-cancerous controls (n=50), of whom 56% were male, mean age of 54y. | (Yang et al., 2015) |
| **EBV RELATED MARKERS** | | | | | | |
| NPC | **IgA VCA + EBV DNA load** | - EBV DNA load combined with IgA VCA into a panel, has a sensitivity of 99% and a specificity of 96–98%. - EBV DNA test is very expensive ($75/test) in comparison to the IgA-VCA test (< $10). Therefore, the latter test can be used in a screening protocol and when positive, followed by an EBV DNA test. | | - Until today, this combination of markers has only been researched in patients with NPC. Other tumour groups should thus be tested in the future. - Quantitative plasma EBV DNA has a low reproducibility. - The predictive value of EBV DNA load is not high enough to perform well in high-risk populations. | - NPC patients (n=139) of whom 74% were male, with mean age of 49y. - Healthy controls (n=178) of whom 26% were male, with a mean age of 52y. - Healthy family members with false positive IgA-VCA (n=36) of whom 39% were male, mean age of 39y. | (Leung et al., 2004) |
| **miRNA** | | | | | | |
| Oral cancer | **Combination of miR-196a and miR-196b** | - Combination of 2 miRNAs with sensitivity and specificity for detecting oral pre-cancer 69% and 85% respectively, and for detecting cancer 88% and 93% respectively. - Minimal invasive and very stable marker in serum and plasma. | | - These markers should be further investigated in large, prospective studies. - The proportion male/female was very different when comparing the oral cancer group with the pre-cancer group and the healthy controls. | - EDTA samples from oral cancer patients (n=90), of whom 91% male and mean age 54y. - Oral pre-cancer patients (n=16) of whom 100% male and mean age 50y. - Healthy controls (n=53) of whom 70% male and mean age 47y. | (Lu et al., 2015) |
| OL, OSCC | **Three-plasma miRNA panel (miR-222-3p, miR-150-5p, and miR-423-5p)** | - There was shown to be a negative correlation of miR-222-3p and miR-423-5p with clinical stage, T stage and lymph node metastasis status, which implies they can be used to monitor tumour progression. - The panel had an area under curve (AUC) of 0.88 for distinguishing OL from OSCC. - It is an easily accessible biomarker (plasma). | | - Almost all the participants are male (241 of 250). - These underlying mechanisms still need to be investigated. | - This study included 250 participants. - Screening and training phase (n=72): normal (n=20), OL (n=20) and OSCC (n=32). - Validation phase (n=178): normal (n=50), OL (n=46) and OSCC (n=82). | (Chang et al., 2018) |
| LSCC | **miR-331-3p, miR-603, miR-1303, miR-660-5p and miR-212-3p individually** | - These stable markers can be measured in a minimally invasive manner (serum, plasma). - This study suggested that these five miRNAs were only expressed in LSCC patients and not in healthy individuals or patients with other diseases. | | - The study group was very small. - The characteristics of the controls and the patients were very different. - The identified miRNAs can only be used for LSCC, although other miRNAs might serve as diagnostic biomarkers for the other HNSCC. | - LSCC patients (n=20) from Mersin, Turkey of whom 90% male and mean age 58.5y. - Unrelated controls (n=44) without history of cancer, chronic degenerative neurological disease, diabetes, hypertension, atopy, autoimmune disease or allergies of whom 45% male and mean age 24.5y. | (Ayaz et al., 2013) |
| Larynx carcinoma | **Combination of hsa-miR-657 and hsa-miR-1287** | - High sensitivity and specificity for the identification of early-stage larynx carcinoma: 100% and 90% in the training samples (n=20) respectively and 86.21% and 100% in the test samples (n=49). | | - These underlying mechanisms still need to be investigated. - Retrospective study design. | - Larynx carcinoma patients of early-stage (n=39). - Healthy controls (n=30). | (Wang et al., 2013) |
| LSCC | **miR-155** | - Upregulated in LSCC tissue and plasma with a sensitivity and specificity of 58.4% and 69.5% respectively for tissue miR-155 and a sensitivity and specificity of 58.4% and 69.5% for plasma miR-155 respectively. Tissue and plasma levels of miR-155 were high in all stages of LSCC (I-IV) making tissue and plasma miR-155 levels a potential diagnostic biomarker for LSCC. - In comparison to the preoperative levels of miR-155, a significant decrease was observed after surgery, pointing to a potential role as prognostic biomarker. | | - Blood miRNAs can be influenced by different clinical and environmental factors like tobacco smoke. The proportion of subjects who smoked was higher in the LSCC population than in the healthy controls. This could partly explain why miRNAs were higher in LSCC patients compared to the control population. - The proportion of men in the LSCC group significantly differed from the control group. | - LSCC patients (n=280) of whom 12.5% were male. - Healthy controls (n=560) of whom 56.3% were male. - Tissue and peripheral blood samples were collected, before and after treatment. | (Wang et al., 2016) |
| **INTERFERONS** | | | | | | |
| OSCC | **Interferon inducible transmembrane protein 1 (IFITM1)** | - Overexpression of IFITM1 in 39% of tumour biopsy OSCC samples. | - It is not clear what causes the variation in the expression level. - There is no evidence for the role of IFITM1 in cancer processes. - Only tested in a high-risk area for OSCC. | | - Indian patients from a Tamil Nadu of Dravidian race with a well differentiated OSCC (n=38). | (Ramanathan and Ramanathan, 2016) |
| OSCC | **ISG15** | - Overexpression of ISG15 in 24 of 30 OSCC samples (80%). | - It is not clear what causes the variation expression level of ISG15. - There is need for a large longitudinal study to see if ISG15 can be useful as a diagnostic biomarker in the clinical setting. - Only tested in a high-risk area for OSCC. | | - Patients with well-differentiated OSCC (n=30) coming from the southern part of India with a similar genetic background. | (Laljee et al., 2013) |
| **OTHERS** | | | | | | |
| OSCC | **Melanoma associated antigens-A (MAGE-A)** | - No expression of these antigens in healthy controls, indicating a high specificity for malignant cells. - The sensitivity of MAGE-A for diagnosis of OSCC rises with multiple MAGE-A expressions. - At least one of the MAGE-A genes is expressed in 81% of stage I and II cases and rises up to 100% in stages III and IV. This means it may be a good biomarker for early diagnosis. | - More studies are needed for determination of the expression profiles of normal oral mucosa close to tumour margins and/or at distance, in order to validate the role of MAGE-A. | | - OSCC patients (n=70); 13 were female and 57 were male. - Patients with normal oral mucosa (n=20). | (Ries et al., 2009) |
| OSCC | **ADAM15, CDC7, IL12RB2 and TNFRSF8** | - ADAM15 and CDC7 were upregulated in OSCC patients. | - The study consists of a rather small sample group. - qPCR validation only showed significant differences for ADAM15 and CDC7. | | - OSCC patients (n=33), 17 men and 16 women   - Discovery set for microarray analysis: n= 5.   - Validation set for qPCR analysis: n= 28. | (Yong-Deok et al., 2015) |
| OSCC | **5-hydroxylmethylcytosine (5-hmC)** | - A gradual loss of 5-hmC from benign dermal nevi to high-grade dysplastic nevi and melanomas was found. - 5-hmC is preserved in reactive lesions and inflammatory lesions, making it easier to distinguish them from dysplastic premalignant oral lesions. | - It remains unclear whether 5-hmC could serve as an accurate gauge of early oncogenesis in difficult and borderline lesions. | | - This study evaluated 66 human cases   - Fibromas (n=9), frictional keratosis (n=9), oral lichen planus (n=10), moderate-to-severe oral epithelial dysplasia (n=15), OSCC (n=23). | (Cuevas-Nunez et al., 2018) |
| HNSCC, especially OPSCC | **Total cfDNA from plasma** | - cfDNA can be obtained in a minimal invasive manner, with quick results and it offers the possibility of repeated analysis. - The total amount of cfDNA was significantly higher in OPSCC patients compared to other HNSCC patients (p = 0.011). | - The total cfDNA was higher in cancer patients compared to healthy controls, however, not statistically significant. - The total cfDNA was higher in clinically advanced disease stages, indicating that this marker is possibly not suited for early detection. - The quality of this study was suboptimal: they had a relatively small study population and the patients were under treatment with chemo- or radiotherapy. | | - HNSCC patients, treated with radiotherapy or chemotherapy but not with surgical excision (n= 27), age: 41.30 ± 7.62y. - Healthy control group (n=15), age: 40.93 ± 8.39y. | (Mazurek et al., 2016) |

**Abbreviations**

EBV = Epstein-Barr virus; HNSCC = head and neck squamous cell carcinoma; LSCC = laryngeal squamous cell carcinoma; miRNA = microRNA; NPC = nasopharyngeal carcinoma; OL = oral leukoplakia; OPSCC = oropharyngeal squamous cell carcinoma; OSCC = oral squamous cell carcinoma.

| **Supplementary table 2: diagnostic biomarkers for head and neck neoplasms – PROTEOMICS** | | | | | | |
| --- | --- | --- | --- | --- | --- | --- |
| **TYPE OF TUMOUR** | **POTENTIAL BIOMARKER** | | **ADVANTAGES** | **RESTRICTIONS** | **STUDY POPULATION** | **REFERENCE** |
| **CYTOKINES** | | | | | | |
| OL with dysplasia | **IL-6** | | - IL-6 levels were found to be significantly higher in patients with tobacco history, leukoplakia and periodontitis. IL-6 levels also increased when dysplasia evolved from mild to severe. This indicates that IL-6 expression can be used as a potential biomarker for lesions at high risk of malignant transformation. - IL-6 levels were found to be 3 to 4 times higher in saliva then in serum, which makes it a great potential salivary biomarker. | - All the patients in the study had periodontitis, which may explain that IL-6 levels were higher than showed in previous studies. - The study didn’t include females, and diet may be a confounding factor. - There is need for large multicenter trials to clarify the role of interleukins in the pathogenesis of oral potentially malignant disorder (OPMD) associated with tobacco. | - 60 patients divided into 3 groups:   - Patients with OL with co-existing periodontitis.   - Periodontitis without OL.   - Healthy controls. - These two last groups were considered control groups. - Patients were in their 3^rd^ to 6^th^ decade of life, regardless of their tobacco history. - All male patients to eliminate difference in gender. | (Sharma et al., 2011) |
| TSCC | **IL-1, IL-6, IL-8, VEGF and TNF-α** | | - IL-1α, IL-6, VEGF-a and TNF-α showed an increase in elevation from controls to TSCC subjects. - IL-1α, IL-6, VEGF-a and TNF-α were highly elevated in TSCC patients with endophytic type. This means that elevation of these cytokines has a correlation with disease severity and mortality. - IL-8 elevation is linked to poor prognosis and increased metastasis. - These are salivary biomarkers and thus easily accessible. | - There is a need for identification of a combination of salivary biomarkers with a good specificity and sensitivity to indicate at-risk lesions and cancerous states. | - 18 TSCC patients divided into 2 different cancer types:   - 8 exophytic.   - 10 endophytic. - The controls were distributed into 4 groups of 14 people: healthy subjects, smoking controls, drinking controls and smoking-drinking controls. | (Korostoff et al., 2011) |
| **OTHERS** | | | | | | |
| OSCC | **RACK1** | - Overexpression of RACK1 is often observed in OSSC cancer cell lines and in OSCC clinical specimens. - There might also be an association with RACK1 levels and prognosis. | | - The involvement of RACK1 in OSCC required for practical application is not clear. Further testing is thus necessary. - The in vivo clinical application of RACK1 siRNA requires further research, as well as the mechanisms of apoptosis induction by RACK1. | - Two-dimensional electrophoresis (2-DE), RT-PCR and Western blotting on OSCC- and matched normal tissues from 10 Chinese patients. - For IHC analyses, 20 normal tissues were collected from healthy persons who went through plastic surgery. - 48 OL and 76 OSCC specimens were collected from the pathology department archives for which a 3-year follow-up was carried out. | (Wang et al., 2008) |
| OSCC and oral epithelial dysplasia lesions | **Phosphorylation of  ribosomal protein s6 (p-RPS6)** | | - This protein was present in all of the oral epithelial dysplasia cases and in 88.68 % of the OSCC patients. RPS6 is thus active in the early events of tumour progression, what makes this a good marker for early detection. | - This marker was only described in carcinomas, not in other histological types of head and neck neoplasms. - It can only be used for the OSCC which developed from oral epithelial dysplasia. The other premalignant lesions developing into OSCC might not involve RPS6. - The study population was quite small. | - 98 paraffin-embedded tissue blocks, collected at the Chulalongkorn University, Bangkok.   - Normal oral mucosa (n=30).   - Epithelial dysplasia (n=15).   - OSCC (n=53). | (Chaisuparat et al., 2013) |
| TSCC | **Adenosine deaminase (ADA)** | | - The study found a significant difference between the salivary ADA levels in TSCC and in the healthy controls. They also found higher levels of serum ADA when staging increased (stage I tot stage III). Thus, ADA might serve as a diagnostic and prognostic biomarker. - It is a salivary biomarker and thus easily accessible. | - When studying enzymes, the outcomes can be controversial. This might be due to the tumour’s histology, stage and therapy, the methods used for collection, and analysis. Enzyme metabolism may also be influenced by smoking, alcohol use, etcetera or by the process of carcinogenesis itself. | - 50 TSCC patients (25 men and 25 women) and 30 healthy controls (15 men and 15 women) from Haryana. - Patients with poor general health, patients who smoked and/or had alcohol abuse were excluded. | (Rai et al., 2011) |
| HNSCC | **Midkine** | | - The sensitivity and specificity of detecting HNSCC, when using 482 pg/mL as the cut-off value, was respectively 57.3% and 85.3%. - There was no link observed between clinical stage and serum midkine concentration. This means that serum midkine is already overexpressed in an early stage of HNSCC. In patients with stage 0-II, overexpression of serum midkine (>482 pg/mL) was found in 44,8%. - There is evidence of midkine as a serum biomarker for prognosis and chemosensitivity in HNSCC. - It is an easily accessible biomarker (serum sample). | - The specificity is rather low. This might be due to the fact that midkine can be increased in several diseases. In this study, they found that its level in individuals over 65 years-old was elevated. Being older of age can be associated with underlying chronic diseases, which might explain the overexpression of midkine. Thus, there is need for studies to focus on the possibility of diverse cut-off values at different age. | - 103 newly diagnosed HNSCC patients (82 men and 21 women) and 116 controls (53 men and 63 women). - The control population consisted of healthy volunteers and individuals with benign otolaryngologic disease. | (Yamashita et al., 2016) |
| HNSCC | **NFκB-p50 and IκBα** | | - This is a serum marker and thus easily accessible. - The p50 level was almost two times as high in HNSCC patients compared to the normal group. - The protein level showed significant correlation with tumour burden, stage and nodal involvement, which could mean that this marker can be used for detection of progression. | - The quality of the study was not optimal: there were more male than female patients included in the study and all the included HNSCC patients were oral cavity patients, oropharynx patients, larynx and nasopharynx patients. The last 2 were significantly less in number, which makes us wonder if this biomarker might be more associated with oral and oropharyngeal cancer. - Most of the patients in the study group presented with late stage HNSCC. A biomarker for diagnosis needs to detect tumours in early stages to improve the prognosis of the patient, so we wonder if these markers are already sufficiently present in early stage HNSCC. | - 104 healthy controls and 125 HNSCC patients (of whom 92% were male) were included:   - Oral cavity cancer patients: 36.8%.   - Oropharyngeal cancer patients: 39.2%.   - Laryngeal cancer patients: 20%.   - Nasopharyngeal cancer patients: 4%. | (Gupta et al., 2015) |
| HNSCC (NPC excluded) | **Salivary total protein + soluble CD44 levels (solCD44)** | | - Detection with the oral rinse test is simple, inexpensive and non-invasive. - The protein levels (total and soluble) were high, regardless of tumour stage or burden. - When having high levels of total protein combined with high levels of solCD44, the risk of HNSCC is nearly 25 times higher than subjects who do not have these elevated levels. | - There is need for a larger matched case-control study because of the small research population. - NPC was excluded. | - Patients with HNSCC (n=102) and healthy controls (n=84), with a similar tobacco and alcohol exposure. - Exclusion of pregnant women, people infected with human immunodeficiency virus (HIV) and NPC, because it tends to behave differently compared to the other HNSCC’s. | (Franzmann et al., 2012) |

**Abbreviations**

HNSCC = head and neck squamous cell carcinoma; NPC = nasopharyngeal carcinoma; OL = oral leukoplakia; OSCC = oral squamous cell carcinoma; TSCC = tongue squamous cell carcinoma.

| **Supplementary table 3: diagnostic biomarkers for head and neck neoplasms - METABOLOMICS** | | | | | |
| --- | --- | --- | --- | --- | --- |
| **TYPE OF TUMOUR** | **POTENTIAL BIOMARKER** | **ADVANTAGES** | **RESTRICTIONS** | **STUDY POPULATION** | **REFERENCE** |
| OSCC | **Altered energy metabolism** (Examples: high glucose, low lactate, low creatine, high creatinine, high choline-creatine ratios etc.). | - This study observed a different energy metabolism in the sera of OSCC patients. They saw a suppressed Krebs cycle, an increased level of ketone bodies and catabolism of protein and amino acids. This is mostly seen in OSCC with later stages, although they found a certain metabolic profile for early stage disease. - It is an easily accessible biomarker (blood sample). | - The study has a small sample size. - It is not clear what the metabolic processes are that lead to this altered energy metabolism, because blood serum consists of various metabolic events in multiple organs. - There is need for further study to assess the specificity of these findings. | - Blood samples from:   - 15 OSCC patients (5 men and 10 women)   - 10 healthy controls (6 men and 4 women) - The patients and healthy controls were all from the United Kingdom. | (Tiziani et al., 2009) |
| OSCC, OPSCC and OL | **Plasma and salivary cortisol levels in the morning** | - The mean plasma cortisol concentration and the salivary levels of cortisol were significantly higher in OSCC patients compared to the OL patients, the risk group and the healthy controls.   In OPSCC patients, the plasma levels were also a lot higher, but not significantly.   - The salivary cortisol levels of OPSCC patients were significantly higher than those in the OL and risk group. - In the OSCC patients, clinical stage had an independent impact on both plasma and saliva cortisol levels. - Saliva and plasma are both easily accessible and great for screening. | - Cortisol is a hormone that is secreted in response to physical or physiological stress. Cancer patients who are already aware of their diagnosis, could potentially experience a great amount of stress, which could influence salivary and serum cortisol levels. - The study group only collected samples in the morning. A more careful evaluation of the HPA-axis could be achieved when analysing the fluctuations of cortisol throughout the day. | - The study included:   - OSCC patients (n=34)   - OPSCC patients (n=17).   - OL patients (n=17).   - Controls (n=52). - The control group consisted of:   - A ‘risk population’: volunteers who had no history of cancer, but who did drink and/or smoke (n=27).   - A ‘healthy population’: volunteers who had no history of cancer, smoking, and/or drinking (n=25). | (Bernabe et al., 2012) |
| OSCC | **A panel of 4 metabolites: choline, betaine, pipecolinic acid, L-carnitine** | - The combination of these 4 biomarkers had an accuracy of 0.997, a sensitivity of 100% and a specificity of 96.7% for OSCC stage I and II in comparison with the control population. Thus, these biomarkers can be used as a biomarker for early diagnosis in OSCC patients. - It is a salivary biomarker and thus easily accessible. | - There is need for larger studies to validate these biomarkers, so they can be clinically used as diagnostic biomarker. | - Saliva samples from:   - 30 OSCC patients (25 men and 5 women).   - 30 healthy controls (25 men and 5 women). - The participants were all Chinese. | (Wang et al., 2014) |
| OSCC | **Plasma levels of nonanoic acid, glucose, galactose, and cysteine + cystine** | - Plasma biomarkers are easily accessible and great for screening. - The altered levels of the metabolites would reflect the energy metabolism of cancer cells and would thus potentially be early detectable   - Levels of glucose (p=0,0008) and galactose (p<0,0001) are significantly lower in OSCC patients when comparing pre- and postoperative plasma samples - Nonanoic acid and cysteine + cystine levels, on the contrary, were higher in the postoperative plasma samples | - This study has a small sample size. | - Plasma samples from:   - 48 OSCC patients (25 men and 23 women).   - 14 patients with oral disease (15 men and 14 women). - The participants were all Japanese. | (Enomoto et al., 2018) |
| OCC | **Salivary glycine and proline** | - The study used a validation and discovery set and thus acquired demonstrated reproducibility. - They are salivary biomarkers and thus easily accessible. | - The study has a small sample size, in particularly the validation set. | - Saliva samples from:   - 79 OCC patients (56 men and 23 women). - 20 healthy controls (12 men and 8 women). | (Lohavanichbutr et al., 2018) |

**Abbreviations**

OCC = oral cavity cancer; OL = oral leukoplakia; OPSCC = oropharyngeal squamous cell carcinoma; OSCC = oral squamous cell carcinoma.

| **Supplementary table 4: diagnostic biomarkers for head and neck neoplasms - GLYCOMICS** | | | | | |
| --- | --- | --- | --- | --- | --- |
| **TYPE OF TUMOUR** | **POTENTIAL BIOMARKER** | **ADVANTAGES** | **RESTRICTIONS** | **STUDY POPULATION** | **REFERENCE** |
| OPC and oral cancer | **total sialic acid/total protein (TSA/TP) ratios and α-l-fucosidase** | - TSA/TP ratios in serum and saliva were significantly increased in OPC and oral cancer patients in comparison to the control population. The same was observed with serum α-l-fucosidase activity. - Salivary and serum TSA/TP ratios and α-l-fucosidase activity progressively increased from controls to OPC patients and to patients with oral cancer. - Salivary levels of these markers were higher than serum levels in subjects from the same group. | - When used separately, these biomarkers had significantly discriminating ability. However, when combined, the specificity of the salivary biomarkers decreased under 50%. - There is need for larger studies to validate these biomarkers and to establish their role in progression of oral cancer. | - The study consisted of 250 subjects:   - 100 healthy controls.   - 50 OPC patients.   - 100 patients with oral cancer. - The majority of the OPC and oral cancer patients were men and chewed tobacco. - Both blood and saliva samples were taken. | (Vajaria et al., 2013) |
| OSCC | **sialic acid, total protein, total sugar** | - The salivary levels of these markers were significantly higher in OSCC patients in comparison to the healthy controls. The levels of free sialic acid, but not protein-bound sialic acid, total protein or total sugar, were also increased in well-differentiated OSCC compared to moderately differentiated OSCC. - Sialic acid is a potential diagnostic biomarker for OSCC. - It is a salivary biomarker and thus easily accessible. | - There is need for a more detailed study with more samples to assess the importance of salivary glycoconjugates as a biomarker of OSCC in clinical use. - The role of total protein and total sugar in malignancy is not clear. - Only tested in a high-risk area for OSCC. | - The study included 60 subjects from a hospital in Dharwad, India:   - 30 OSCC patients.   - 30 healthy controls. | (Sanjay et al., 2008) |

**Abbreviations**

OPC = oropharyngeal cancer; OSCC = oral squamous cell carcinoma.

| **Supplementary table 5: diagnostic biomarkers for head and neck neoplasms – OTHERS** | | | | | |
| --- | --- | --- | --- | --- | --- |
| **TYPE OF TUMOUR** | **POTENTIAL BIOMARKER** | **ADVANTAGES** | **RESTRICTIONS** | **STUDY POPULATION** | **REFERENCE** |
| **THYROID CANCER** | | | | | |
| PTC | **Higher mean platelet volume (MPV)** | - This is an easily accessible biomarker (blood sample). - When compared to benign goiter patients and healthy controls, the preoperative MPV levels in patients with PTC were found to be significantly higher. | - This marker has only been studied for PTC. - The quality of the study was not optimal: the study was retrospective and had a relatively small sample size. | - PTC patients (n=66), age=42.0 (24.0−64.0). - Benign goiter patients (n=32), age= 45.0 (19.0−76.0). - Healthy controls (n=28), age= 37.6 (27.0−50.0). - The proportion male/female in the 3 groups was quite similar (15-21% male). | (Baldane et al., 2015) |

**Abbreviations**

PTC = papillary thyroid carcinoma.

AYAZ, L., GORUR, A., YAROGLU, H. Y., OZCAN, C. & TAMER, L. 2013. Differential expression of microRNAs in plasma of patients with laryngeal squamous cell carcinoma: potential early-detection markers for laryngeal squamous cell carcinoma. *J Cancer Res Clin Oncol,* 139**,** 1499-506.

BALDANE, S., IPEKCI, S. H., SOZEN, M. & KEBAPCILAR, L. 2015. Mean platelet volume could be a possible biomarker for papillary thyroid carcinomas. *Asian Pac J Cancer Prev,* 16**,** 2671-4.

BERNABE, D. G., TAMAE, A. C., MIYAHARA, G. I., SUNDEFELD, M. L., OLIVEIRA, S. P. & BIASOLI, E. R. 2012. Increased plasma and salivary cortisol levels in patients with oral cancer and their association with clinical stage. *J Clin Pathol,* 65**,** 934-9.

CHAISUPARAT, R., ROJANAWATSIRIVEJ, S. & YODSANGA, S. 2013. Ribosomal protein S6 phosphorylation is associated with epithelial dysplasia and squamous cell carcinoma of the oral cavity. *Pathol Oncol Res,* 19**,** 189-93.

CHANG, Y. A., WENG, S. L., YANG, S. F., CHOU, C. H., HUANG, W. C., TU, S. J., CHANG, T. H., HUANG, C. N., JONG, Y. J. & HUANG, H. D. 2018. A Three-MicroRNA Signature as a Potential Biomarker for the Early Detection of Oral Cancer. *Int J Mol Sci,* 19.

CUEVAS-NUNEZ, M. C., GOMES, C. B. F., WOO, S. B., RAMSEY, M. R., CHEN, X. L., XU, S., XU, T., ZHAN, Q., MURPHY, G. F. & LIAN, C. G. 2018. Biological significance of 5-hydroxymethylcytosine in oral epithelial dysplasia and oral squamous cell carcinoma. *Oral Surg Oral Med Oral Pathol Oral Radiol,* 125**,** 59-73 e2.

ENOMOTO, Y., KIMOTO, A., SUZUKI, H., NISHIUMI, S., YOSHIDA, M. & KOMORI, T. 2018. Exploring a Novel Screening Method for Patients with Oral Squamous Cell Carcinoma: A plasma Metabolomics Analysis. *Kobe J Med Sci,* 64**,** E26-e35.

FRANZMANN, E. J., REATEGUI, E. P., PEREIRA, L. H., PEDROSO, F., JOSEPH, D., ALLEN, G. O., HAMILTON, K., REIS, I., DUNCAN, R., GOODWIN, W. J., HU, J. J. & LOKESHWAR, V. B. 2012. Salivary protein and solCD44 levels as a potential screening tool for early detection of head and neck squamous cell carcinoma. *Head Neck,* 34**,** 687-95.

GUERRERO-PRESTON, R., SOUDRY, E., ACERO, J., ORERA, M., MORENO-LOPEZ, L., MACIA-COLON, G., JAFFE, A., BERDASCO, M., ILI-GANGAS, C., BREBI-MIEVILLE, P., FU, Y., ENGSTROM, C., IRIZARRY, R. A., ESTELLER, M., WESTRA, W., KOCH, W., CALIFANO, J. & SIDRANSKY, D. 2011. NID2 and HOXA9 promoter hypermethylation as biomarkers for prevention and early detection in oral cavity squamous cell carcinoma tissues and saliva. *Cancer Prev Res (Phila),* 4**,** 1061-72.

GUPTA, A., KUMAR, R., SAHU, V., AGNIHOTRI, V., SINGH, A. P., BHASKER, S. & DEY, S. 2015. NFkappaB-p50 as a blood based protein marker for early diagnosis and prognosis of head and neck squamous cell carcinoma. *Biochem Biophys Res Commun,* 467**,** 248-53.

KOROSTOFF, A., REDER, L., MASOOD, R. & SINHA, U. K. 2011. The role of salivary cytokine biomarkers in tongue cancer invasion and mortality. *Oral Oncol,* 47**,** 282-7.

LALJEE, R. P., MUDDAIAH, S., SALAGUNDI, B., CARIAPPA, P. M., INDRA, A. S., SANJAY, V. & RAMANATHAN, A. 2013. Interferon stimulated gene-ISG15 is a potential diagnostic biomarker in oral squamous cell carcinomas. *Asian Pac J Cancer Prev,* 14**,** 1147-50.

LEUNG, S. F., TAM, J. S., CHAN, A. T., ZEE, B., CHAN, L. Y., HUANG, D. P., VAN HASSELT, A., JOHNSON, P. J. & LO, Y. M. 2004. Improved accuracy of detection of nasopharyngeal carcinoma by combined application of circulating Epstein-Barr virus DNA and anti-Epstein-Barr viral capsid antigen IgA antibody. *Clin Chem,* 50**,** 339-45.

LOHAVANICHBUTR, P., ZHANG, Y., WANG, P., GU, H., NAGANA GOWDA, G. A., DJUKOVIC, D., BUAS, M. F., RAFTERY, D. & CHEN, C. 2018. Salivary metabolite profiling distinguishes patients with oral cavity squamous cell carcinoma from normal controls. *PLoS One,* 13**,** e0204249.

LU, Y. C., CHANG, J. T., HUANG, Y. C., HUANG, C. C., CHEN, W. H., LEE, L. Y., HUANG, B. S., CHEN, Y. J., LI, H. F. & CHENG, A. J. 2015. Combined determination of circulating miR-196a and miR-196b levels produces high sensitivity and specificity for early detection of oral cancer. *Clin Biochem,* 48**,** 115-21.

MAZUREK, A. M., RUTKOWSKI, T., FISZER-KIERZKOWSKA, A., MALUSECKA, E. & SKLADOWSKI, K. 2016. Assessment of the total cfDNA and HPV16/18 detection in plasma samples of head and neck squamous cell carcinoma patients. *Oral Oncol,* 54**,** 36-41.

PUTTIPANYALEARS, C., ARAYATAWEEGOOL, A., CHALERTPET, K., RATTANACHAYOTO, P., MAHATTANASAKUL, P., TANGJATURONSASME, N., KEREKHANJANARONG, V., MUTIRANGURA, A. & KITKUMTHORN, N. 2018. TRH site-specific methylation in oral and oropharyngeal squamous cell carcinoma. *BMC Cancer,* 18**,** 786.

RAI, B., KAUR, J., JACOBS, R. & ANAND, S. C. 2011. Adenosine deaminase in saliva as a diagnostic marker of squamous cell carcinoma of tongue. *Clin Oral Investig,* 15**,** 347-9.

RAMANATHAN, A. & RAMANATHAN, A. 2016. Interferon Induced Transmembrane Protein-1 Gene Expression as a Biomarker for Early Detection of Invasive Potential of Oral Squamous Cell Carcinomas. *Asian Pac J Cancer Prev,* 17**,** 2297-9.

RIES, J., MOLLAOGLU, N., TOYOSHIMA, T., VAIRAKTARIS, E., NEUKAM, F. W., PONADER, S. & NKENKE, E. 2009. A novel multiple-marker method for the early diagnosis of oral squamous cell carcinoma. *Dis Markers,* 27**,** 75-84.

SANJAY, P. R., HALLIKERI, K. & SHIVASHANKARA, A. R. 2008. Evaluation of salivary sialic acid, total protein, and total sugar in oral cancer: a preliminary report. *Indian J Dent Res,* 19**,** 288-91.

SHARMA, M., BAIRY, I., PAI, K., SATYAMOORTHY, K., PRASAD, S., BERKOVITZ, B. & RADHAKRISHNAN, R. 2011. Salivary IL-6 levels in oral leukoplakia with dysplasia and its clinical relevance to tobacco habits and periodontitis. *Clin Oral Investig,* 15**,** 705-14.

SUSHMA, P. S., JAMIL, K., KUMAR, P. U., SATYANARAYANA, U., RAMAKRISHNA, M. & TRIVENI, B. 2016. PTEN and p16 genes as epigenetic biomarkers in oral squamous cell carcinoma (OSCC): a study on south Indian population. *Tumour Biol,* 37**,** 7625-32.

TIZIANI, S., LOPES, V. & GUNTHER, U. L. 2009. Early stage diagnosis of oral cancer using 1H NMR-based metabolomics. *Neoplasia,* 11**,** 269-76, 4p following 269.

VAJARIA, B. N., PATEL, K. R., BEGUM, R., SHAH, F. D., PATEL, J. B., SHUKLA, S. N. & PATEL, P. S. 2013. Evaluation of serum and salivary total sialic acid and alpha-l-fucosidase in patients with oral precancerous conditions and oral cancer. *Oral Surg Oral Med Oral Pathol Oral Radiol,* 115**,** 764-71.

WANG, J. L., WANG, X., YANG, D. & SHI, W. J. 2016. The Expression of MicroRNA-155 in Plasma and Tissue Is Matched in Human Laryngeal Squamous Cell Carcinoma. *Yonsei Med J,* 57**,** 298-305.

WANG, Q., GAO, P., WANG, X. & DUAN, Y. 2014. Investigation and identification of potential biomarkers in human saliva for the early diagnosis of oral squamous cell carcinoma. *Clin Chim Acta,* 427**,** 79-85.

WANG, Y., CHEN, M., TAO, Z., HUA, Q., CHEN, S. & XIAO, B. 2013. Identification of predictive biomarkers for early diagnosis of larynx carcinoma based on microRNA expression data. *Cancer Genet,* 206**,** 340-6.

WANG, Z., JIANG, L., HUANG, C., LI, Z., CHEN, L., GOU, L., CHEN, P., TONG, A., TANG, M., GAO, F., SHEN, J., ZHANG, Y., BAI, J., ZHOU, M., MIAO, D. & CHEN, Q. 2008. Comparative proteomics approach to screening of potential diagnostic and therapeutic targets for oral squamous cell carcinoma. *Mol Cell Proteomics,* 7**,** 1639-50.

YAMASHITA, T., SHIMADA, H., TANAKA, S., ARAKI, K., TOMIFUJI, M., MIZOKAMI, D., TANAKA, N., KAMIDE, D., MIYAGAWA, Y., SUZUKI, H., TANAKA, Y. & SHIOTANI, A. 2016. Serum midkine as a biomarker for malignancy, prognosis, and chemosensitivity in head and neck squamous cell carcinoma. *Cancer Med,* 5**,** 415-25.

YANG, X., DAI, W., KWONG, D. L., SZETO, C. Y., WONG, E. H., NG, W. T., LEE, A. W., NGAN, R. K., YAU, C. C., TUNG, S. Y. & LUNG, M. L. 2015. Epigenetic markers for noninvasive early detection of nasopharyngeal carcinoma by methylation-sensitive high resolution melting. *Int J Cancer,* 136**,** E127-35.

YONG-DEOK, K., EUN-HYOUNG, J., YEON-SUN, K., KANG-MI, P., JIN-YONG, L., SUNG-HWAN, C., TAE-YUN, K., TAE-SUNG, P., SOUNG-MIN, K., MYUNG-JIN, K. & JONG-HO, L. 2015. Molecular genetic study of novel biomarkers for early diagnosis of oral squamous cell carcinoma. *Med Oral Patol Oral Cir Bucal,* 20**,** e167-79.
